# Supplementary material for: Non-uniform temporal scaling of developmental processes in the mammalian cortex
Source: Nat Commun. 2023 Sep 23;14:5950. doi: 10.1038/s41467-023-41652-5 (PMC10517946; doi:10.1038/s41467-023-41652-5)
Supplement: Supplementary file 1 — Supplementary Information [file 41467_2023_41652_MOESM1_ESM.pdf]

SUPPLEMENTARY FIGURES

a

Stage	Mouse	Dunnart
18	E 10.5	P 0-3
19	E 11.5	P 4-7
20	E 12.5	P 8-11
21	E 13.5	P 12-16
22	E 14.5	P 17-19
23	E 15.5	P 20-23
24	E 16.5	P 24-26
25	E 17.5	P 27-30
26	E 18.5	P 31-35
27	P0-P4	P 36-40
28	P4-P10	P 41-50

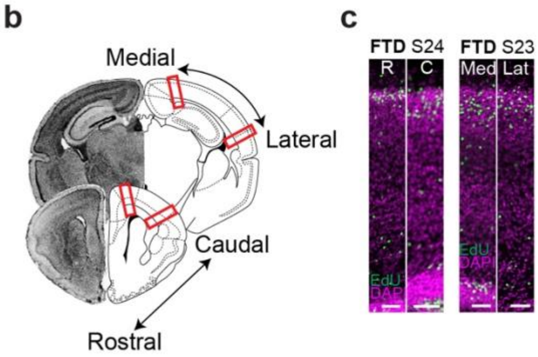

Supplementary Fig. 1: Equivalent staging system of mice and dunnarts and supplementary neurogenic gradient data.

(a) Staging system summarized from[1]. This staging system was developed based on body and head morphological features, independent of cortical formation.

(b) Schematic of position of cortical images taken for rostrocaudal/mediolateral EdU cell birth gradient determination. Figure adapted with modification from Suárez et al., 2018 *Proc Natl Acad Sci U S A* 115, 9622-9627 (ref [2]).

(c) Representative cortical images of the oldest ages quantified for rostral versus caudal (left) and medial versus lateral (right) EdU quantification.

C, caudal; E, embryonic day; FTD, fat-tailed dunnart; Lat, lateral; Med, medial; P, postnatal day; R, rostral; S, stage. Scale bars: c = 50 µm.

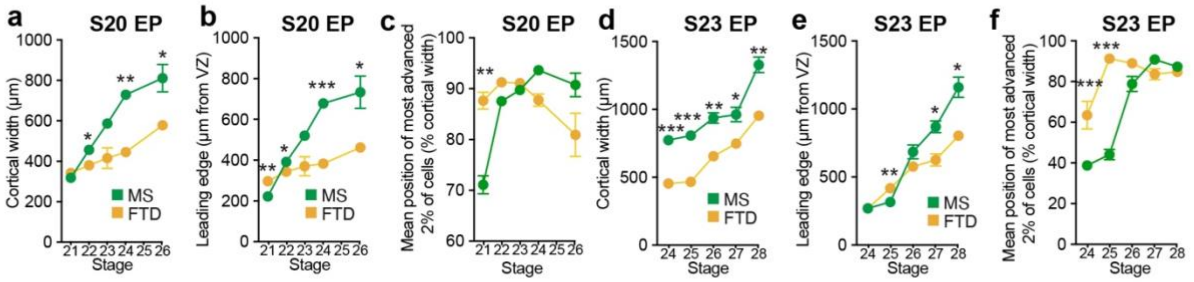

Supplementary Fig. 2: Additional quantifications related to migration by stage comparison between species.

(a-f) Additional quantifications from Fig. 2 showing the average cortical width at successive stages after electroporation (a,d), the absolute distance of the leading edge of the wave of migratory cells (b,e) and the mean position of the most advanced 2% of TdTom^{+} cells (c,f) following S20 DL electroporation (a-c) and S23 UL electroporation (d-f).

N for MS in (a-c) S21:5, S22:5, S23:9, S24:3, S26:3; FTD in (a-c) S21:21, S22:13, S23:3, S24:3, S26:3. For MS in (d-f) S24:9, S25:8, S26:5, S27:4, S28:4; FTD (d-f) S24:7, S25:9, S26:4, S27:5, S28:7.

Data are presented as mean values +/- SEM and were compared using pairwise *t*-tests (a,b,d,e) or Mann-Whitney *U* tests (c,f); *$p < 0.05$, **$p < 0.01$, ***$p < 0.001$. See Supplementary Table 3 for exact *p* values and statistical test details.

EP, electroporation; FTD, fat-tailed dunnart; MS, mouse; S, stage; VZ, ventricular zone.

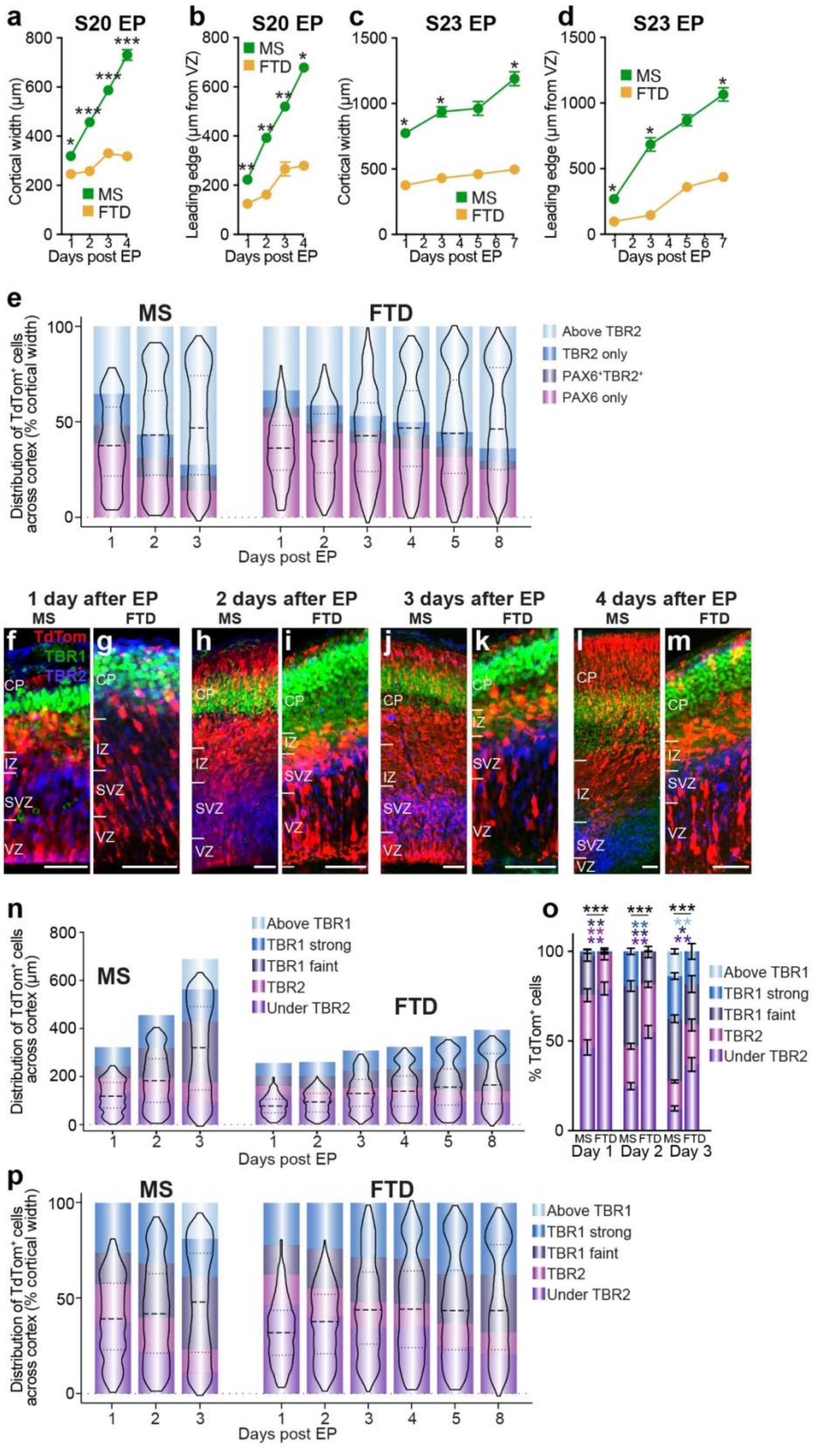

Supplementary Fig. 3: Additional quantifications related to migration by day comparison between species. (a-d) Additional quantifications from Fig. 3 of cortical width (a,c) and absolute distance of the leading edge of the migratory wave from the VZ (b,d) of mice and dunnarts electroporated at S20 (a-b) or S23 (c-d) and collected 1-7 days later. N for MS in (a,b) Day1:5, Day2:5, Day3:9, Day4:3; FTD in (a,b) Day1:7, Day2:8, Day3:6, Day4:8. For MS in (c,d) Day1:9, Day3:5, Day5:4, Day7:9; FTD in (c,d) Day1:3, Day3:4, Day5:3, Day7:3.

(e) Violin plots showing TdTom$^+$ cells distribution in mouse and dunnart cortices following S20 electroporation and collection across subsequent days, represented as percentage of total average cortical width. TBR2 and PAX6 immunolabeling demarcates the borders of cortical compartments. N for MS, Day1:5; Day2:7; Day3:16; FTD, Day1:11; Day2:13; Day3:13; Day4:9; Day5:12; Day8:12.

(f-m) Mice (f,h,j,l) and dunnarts (g,i,k,m) electroporated with CAG-TdTom at S20 and collected 1-4 days later and immunolabeled for TBR1 and TBR2.

(n) Violin plots of TdTom$^+$ cell distribution from f-m showing progressive distribution overlying bands demarcated by TBR2 and TBR1 expression. N for MS, Day1:5; Day2:8; Day3:9; FTD Day1:8; Day2:9; Day3:7; Day4:7; Day5:9; Day8:7.

(o) Quantification of TdTom$^+$ cells proportion in each immunolabeled band from 1-3 days post-electroporation at S20. N for MS Day1:6, Day2:7, Day3:13; FTD Day1:9, Day2:12, Day3:8.

(p) Violin plots based on the same data displayed in (n), represented as a percentage of total cortical width.

Data are presented as mean values +/- SEM in all the graphs, except for (e,n,p), which are presented as the median with the first and third quartiles, and were compared using pairwise *t*-test (a) or Mann-Whitney *U* tests (b,c,d) or Npmv followed by pairwise log ratio comparisons (o); $*p < 0.05$, $**p < 0.01$, $***p < 0.001$. See Supplementary Table 3 for exact *p* values and statistical test details.

CP, cortical plate; DL, deep layers; EP, electroporation; FTD, fat-tailed dunnart; IZ, intermediate zone; MS, mouse; SVZ, subventricular zone; UL, upper layers; VZ, ventricular zone. Scale bars: f-m = 50 µm.

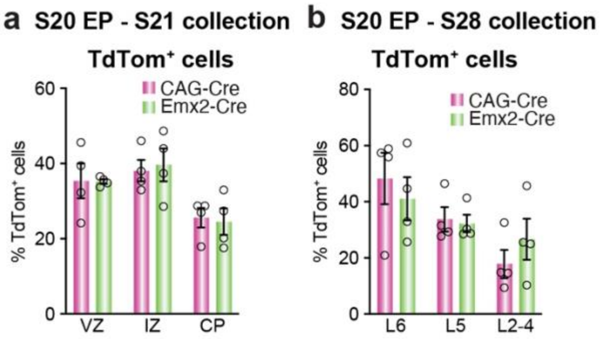

Supplementary Fig. 4: TdTom$^+$ cells are not differentially localized between CAG-Cre and Emx2-Cre conditions.

(a) Quantification of the proportion of CAG-TdTom$^+$ cells (expression not driven by Cre) following S20 (P11) electroporation and S21 (P16) collection of dunnart cortex under CAG-Cre and Emx2-Cre conditions.

(b) Quantification of the proportion of CAG-TdTom$^+$ cells (expression not driven by Cre) following S20 (P11) electroporation and S28 (P50) collection of dunnart cortex under CAG-Cre and Emx2-Cre conditions. N:4 of biologically independent animals in both the CAG-Cre and Emx2-Cre groups. Data are presented as mean values +/- SEM and were compared using MANOVA followed by log ratio comparisons (a,b); *$p < 0.05$, **$p < 0.01$, ***$p < 0.001$. See Supplementary Table 3 for exact p values and statistical test details.

CP, cortical plate; IZ, intermediate zone; L, layer; S, stage; VZ, ventricular zone.

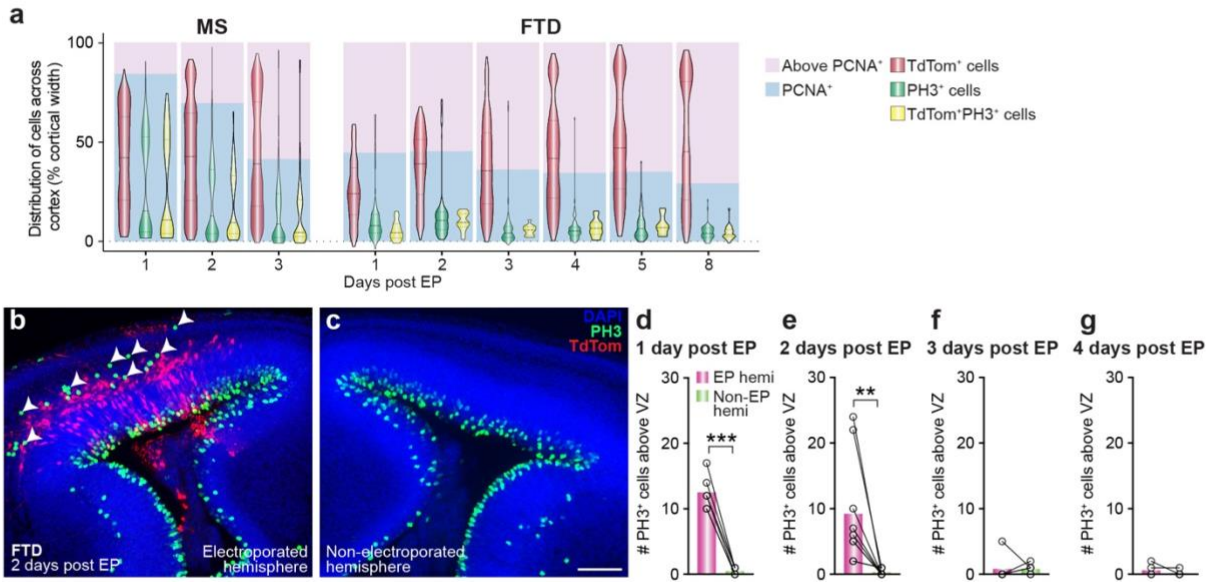

Supplementary Fig. 5: The electroporated hemisphere of dunnarts contains more subventricular mitosing cells than the non-electroporated hemisphere in the first two days post-electroporation.

(a) Violin plots showing aggregates of brains electroporated with TdTom and collected between 1 and 8 days later for mice (left) and dunnarts (right), normalized to total cortical width. Separate distributions of cells across the cortical width are shown for all TdTom$^+$ cells, PH3$^+$ cells and TdTom$^+$PH3$^+$ cells. The averaged boundaries of the PCNA$^+$ and PCNA$^-$ regions as a percentage of the total cortical width are shown for each age underneath the plots. N for MS Day1:6; Day2:7; Day3:13; FTD Day1:9; Day2:12; Day3:9; Day4:9; Day5:10; Day8:15.

(b,c) Images of a single dunnart brain collected two days after a S20 electroporation, showing both the CAG-TdTom electroporated hemisphere (b) and the non-electroporated hemisphere (c) with DAPI and PH3 immunolabeling.

(d-f) Quantification of the number of PH3$^+$ cells above the ventricular zone in the whole cortical section of the electroporated and non-electroporated hemispheres for animals collected 1-4 days after S20 CAG-TdTom electroporation. N for analysis in (d): 6; (e): 9; (f): 6; (g): 5.

Data are presented as mean values +/- SEM in all the graphs, except (a), which is presented as the median with the first and third quartiles, and were compared using *t*-tests (b,f) or Wilcoxon signed-rank test (e,g); $^*p < 0.05$, $^{**}p < 0.01$, $^{***}p < 0.001$. See Supplementary Table 3 for exact *p* values and statistical test details. EP, electroporation; FTD, fat-tailed dunnart; hemi, hemisphere; MS, mouse; VZ, ventricular zone. Scale bars: b,c = 100 µm.

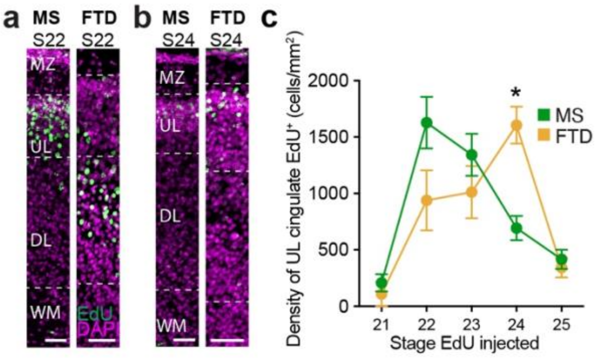

Supplementary Fig. 6: The projection population (upper layers) of the cingulate cortex is born later in dunnarts than in mice.

(a,b) Cingulate cortex images following EdU injection at S22 (a) or S24 (b) and collection at S27.

(c) Quantification of upper layer cingulate EdU^+ cell density in mouse or dunnart brains across 5 ages of EdU injection collected at S27. N for MS S21:4, S22:4, S23:4, S24:4, S25:4; FTD S21:4, S22:3, S23:4, S24:4, S25:3. Data are presented as mean values +/- SEM and were all compared with paired *t*-test); $*p < 0.05$, $**p < 0.01$, $***p < 0.001$. See Supplementary Table 3 for exact *p* values and statistical test details.

DL, deep layers; FTD, fat-tailed dunnart; MS, mouse; MZ, marginal zone; S, stage; UL, upper layers; WM, white matter. Scale bars: a,b = 50 µm.

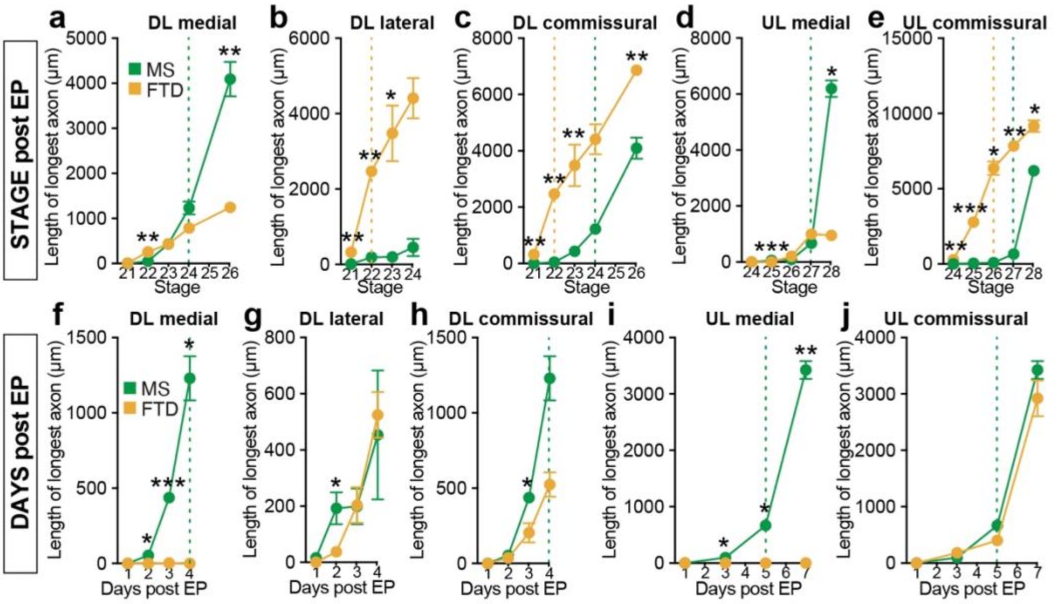

Supplementary Fig. 7: Non-normalized graphs of axon extension by stage and by day.

(a-e) Quantifications relating to Fig. 7 of axon length compared between mice and dunnarts collected at subsequent stages following S20 DL electroporation (a-c) and S23 UL electroporation (d,e) representing the absolute length of axons (not normalized to cortical width). Dotted lines represent the stage of midline crossing (where relevant to axonal population) for mice (green) or dunnarts (orange). N for MS in (a-c) S21:5, S22:6, S23:10, S24:3, S26:6; FTD in (a- c) S21:21, S22:7, S23:3, S24:4, S26:6. N for MS in (d,e) S24:9, S25:7, S26:5, S27:5, S28:5; FTD in (d,e) S24:8, S25:13, S26:4, S27:6, S28:5.

(f,j) Quantifications relating to Fig. 7 of axon length compared between mice and dunnarts collected on subsequent days following S20 DL electroporation (a-c) and S23 UL electroporation (d,e), representing the absolute length of axons (not normalized to cortical width). Dotted lines represent the stage of midline crossing (where relevant to axonal population) for mice (green) or dunnarts (orange). N for MS in (f-h) Day1:5, Day2:6, Day3:10, Day4:3; FTD in (f-h) Day1:7, Day2:7, Day3:10, Day4:4. N for MS in (i,j) Day1:9, Day3:5, Day5:5, Day7:9; FTD in (i,j) Day1:3, Day3:5, Day5:4, Day7:6.

Data are presented as mean values +/- SEM. All data were compared with pairwise Mann-Whitney U tests; **p* < 0.05, ***p* < 0.01, ****p* < 0.001. See Supplementary Table 3 for exact *p* values and statistical test details. DL, deep layers; EP, electroporation; FTD, fat-tailed dunnart; MS, mouse; UL, upper layers.

SUPPLEMENTARY METHODS

**Plasmid**	**Concentration**	**Source**
CAG-TdTomato	1 µg/µl (except Fig. 4a-c 0.5 µg/µl)	Clontech TdTomato fluorophore subcloned into CAG in the laboratory of Linda Richards
CAG-eYFP	1 µg/µl	Gift from Tetsuichiro Saito
CAG-Cre	0.1 µg/µl	Gift from Fernando García-Moreno [3]
Emx2-Cre	0.1 µg/µl	Gift from Fernando García-Moreno [3]
CAG-FloxedStop-GFP	1 µg/µl	Gift from Hiroshi Kawasaki [4]
PBCAG-STOP-H2BEGFP	0.5 µg/µl	Gift from Fernando García-Moreno [3]

Supplementary Table 1: Plasmid concentrations and sources.

**Primary antibodies**	**Company and catalog number**	**Concentration**
Rat anti-CTIP2	Abcam ab18465	1:500
Mouse anti-NEUROD1	Abcam ab60704	1:400
Rabbit anti-PAX6	Sigma-Aldrich ab2237	1:250
Mouse anti-PCNA	SantaCruz sc56	1:250
Rabbit anti-PH3	CST 533485	1:500
Rabbit anti-SATB2	Abcam ab69995	1:500
Rabbit anti-SOX2	Merck ab5603	1:500
Rabbit anti-TBR1	Abcam ab31940	1:500
Chicken anti-TBR2 (Fig. 3c-j; S3e-p)	Millipore ab15894	1:1000
Sheep anti-TBR2 (Fig. 5j-o)	Invitrogen PA5-47818	1:400
Goat anti-TdTomato	Sicgen AB8181-200	1:1000
Chicken anti-GFP	Abcam ab13970	1:750
**Secondary antibodies**	**Company and catalog number**	**Concentration**
Donkey anti-chicken biotinylated	Millipore AP194B	1:500
Donkey anti-goat 555	Invitrogen A32816	1:500
Donkey anti-rabbit 488	Thermofisher A21206	1:500
Donkey anti-mouse biotinylated	Jackson 715-065-151	1:500
Donkey anti-goat biotinylated	Jackson 705-065-147	1:500
Donkey anti-rat biotinylated	Jackson 712-065-153	1:500
Streptavidin-647	Jackson 16600084	1:500

Supplementary Table 2: Primary and secondary antibody catalog number and concentration.

Figure 1				
**Panel**	**Groups**	**Parametric**	**Omnibus**	**Post-hoc/Pairwise tests**
**b**	One between-subjects factor: Stage injected (4 levels). The log2 EdU density ratio of rostral over caudal. Samples (FTD only).<br>S20: 3<br>S21: 4<br>S23: 3<br>S24: 4	Yes.<br>Shapiro-Wilk (W =0.9502, p = 0.56345). ns<br>Levene's test (W = 1.444, p = 0.288). ns	One-way ANOVA (F(3,10) = 13.6963, p = 0.00071). ***	Pairwise t-tests:<br>20-21: t(2.59) = 2.88, p = 0.113. ns<br>20-23: t(4.0) = 3.83, p = 0.096. ns<br>20-24: t(2.05) = 4.17, p = 0.101. ns<br>21-23: t(4.7) = 1.87, p = 0.149. ns<br>21-24: t(6.0) = 2.78, p = 0.095. ns<br>23-24: t(2.31) = 0.18, p = 0.87. ns
**c**	One between-subjects factor: Stage injected (3 levels). The log2 EdU density ratio of medial over lateral. Samples (FTD only).<br>S20: 3<br>S21: 4<br>S23: 3	Yes.<br>Shapiro-Wilk (W = 0.894, p = 0.1873). ns<br>Levene's test (W = 0.3553, p = 0.713). ns	One-way ANOVA (F(2,7) = 13.470, p = 0.003984). **	Pairwise t-tests:<br>20-21: t(3.052) = -2.87, p = 0.074. ns<br>20-23: t(4.0) = -4.40, p = 0.035. *<br>21-23: t(3.023) = -2.68, p = 0.074. ns
**g**	Comparisons between species (2 species) at each stage (8 stages). The weighted median of EdU$^+$cells across cortex. Samples (MS/FTD):<br>S18: 6/4<br>S19: 6/5<br>S20: 6/4<br>S21: 6/6<br>S22: 6/3<br>S23: 6/5<br>S24: 6/4<br>S25: 6/3	No.<br>Shapiro-Wilk (W = 0.795, p < 0.001). ***<br>Levene's test (W = 2.85, p = 0.0017). **	NA	Pairwise Mann-Whitney U tests:<br>S18: U = 8.0, p = 0.544. ns<br>S19: U = 15.0, p = 1.0. ns<br>S20: U = 22.0, p = 0.102. ns<br>S21: U = 29.0, p = 0.149. ns<br>S22: U = 18.0, p = 0.0952. ns<br>S23: U = 29.0, p = 0.069. ns<br>S24: U = 21.0, p = 0.133. ns<br>S25: U = 14.0, p = 0.35. ns
**h**	Two between-subjects factors: Stage (8 levels), Species (2 levels). The log ratio of EdU$^+$cells in upper layers over deep layers. Samples same as g	Yes.<br>Shapiro-Wilk (W = 0.9883, p = 0.6586). ns	NA	Pairwise t-tests:<br>S18: t(6.293) = -2.1096, p = 0.156. ns<br>S19: t(8.767) = -2.8968, p = 0.04837. *<br>S20: t(4.489) = 1.0723, p = 0.3576. ns<br>S21: t(10) = 1.234, p = 0.327. ns<br>S22: t(4.75) = 9.657, p = 0.00211. **<br>S23: t(8.989) = 4.958, p = 0.00314. **<br>S24: t(7.76) = 1.679, p = 0.2125. ns<br>S25: t(4.093) = 1.04, p = 0.356. ns
**i**	Two between-subjects factors: Stage (8 levels), Species (2 levels). EdU$^+$cell density. Samples same as g	No.<br>Shapiro-Wilk (W = 0.965, p = 0.0232). *	NA	Pairwise Mann-Whitney U tests:<br>S18: U = 6, p = 0.3492. ns<br>S19: U = 11, p = 0.6135. ns<br>S20: U = 24, p = 0.0254. *<br>S21: U = 20, p = 0.818. ns<br>S22: U = 1, p = 0.0952. ns<br>S23: U = 0, p = 0.0254. *<br>S24: U = 0, p =0.0254. *<br>S24: U = 4, p = 0.3492. ns
**l**	One between-subjects factor: Species (2 levels). Adult cortical width. Samples (MS/FTD): 5/5	Yes.<br>Shapiro-Wilk: (W = 0.938, p = 0.531). ns	NA	Independent samples t-test (two-sided): t(8) = 17.233, p < 0.0001. ***
**m**	One between-subjects factor: Species (2 levels). Multivariate DVs (IZ, L6, L5, L2/4, MZ). Omnibus testing on isometric log ratio transforms.	Yes.<br>Henze-Zirkler (HZ = 0.573, p = 0.667). ns<br>Box's M (Chi2(10) = 11.822, p = 0.297). ns	MANOVA.<br>Wilk's Lambda (F(4,5) = 8.3789, p = 0.0193). *	Log ratio comparisons:<br>IZ/VZ: p > 0.05<br>L6: p < 0.01. **<br>L5: p < 0.05. *<br>L2/4: p < 0.05. *<br>MZ: p < 0.05. *
**n**	Two between-subjects factors: Species (2 levels), Layer (5 levels). The density of DAPI$^+$ cells. Samples (MS/FTD): 5/5	No.<br>Shapiro-Wilk (W = 0.928, p = 0.005). **<br>Levene's test (W = 0.666, p = 0.734). ns	Aligned Rank Test.<br>Main effect of Species (F(1,40) = 33.968, p < 0.0001). Main effect of Layer (F(4,40) = 24.236, p < 0.0001). *** Species*Layer interaction (F(4,40) = 5.785, p = 0.0009). **	Pairwise Mann-Whitney U tests.<br>IZ: U = 0.0, p = 0.020. *<br>L6: U = 0.0, p = 0.020. *<br>L5: U = 14.0, p = 0.841. ns<br>L24: U = 6.0, p = 0.370. ns<br>MZ: U = 7.0, p = 0.387. ns

Figure 2				
**Panel**	**Groups**	**Parametric**	**Omnibus**	**Post-hoc/Pairwise tests**
**b**	Two between-subjects factors: Species (2 levels), Stage (5 levels). Examining the leading edge of deep layer TdTom$^+$ cells normalized to the cortical width. Samples (MS/FTD):<br>S21: 5/20<br>S22: 5/13<br>S23: 9/3<br>S24: 3/3<br>S26:3/3	No.<br>Shapiro-Wilk (W = 0.88024, p < 0.0001). ***	NA	Pairwise Mann-Whitney U tests:<br>S21: U = 6.0, p = 0.000753. ***<br>S22: U = 7.0, p = 0.0245. *<br>S23: U = 12.0, p = 0.864. ns<br>S24: U = 9.0, p = 0.125. ns<br>S26: U = 9.0, p = 0.125. ns
**d**	Two between-subjects factors: Species (2 levels), Stage (5 levels). Examining the leading edge of upper layer TdTom$^+$ cells	No.<br>Shapiro-Wilk (W = 0.928, p < 0.00138). **	NA	Pairwise Mann-Whitney U tests:<br>S24: U = 0.0, p = 0.000437. ***<br>S25: U = 0.0, p = 0.000411. ***

	normalized to the cortical width. Samples (MS/FTD):<br>S24: 9/7<br>S25: 8/9<br>S26: 5/4<br>S27: 4/5<br>S28: 4/7	Levene's test (F(9,52) = 2.5892, p = 0.01513). *		S26: U = 2.0, p = 0.07937. ns<br>S27: U = 19.0, p = 0.0529. ns<br>S28: U = 32.0, p = 0.230. ns
**e, S21**	One between-subjects factor: Species (2 levels). Multivariate DVs: UL, DL, IZ, VZ. Omnibus tests on isometric log ratio transformed data. Samples (MS/FTD): 5/21	No.<br>Henze-Zirkler (MS: HZ = 0.4161, p = 0.483; FTD: HZ = 2.6577, p < 0.0001). ***<br>Box's M (Chi2(6) = 8.382, p = 0.2114). ns	Npmv:<br>Wilks' Lambda<br>W(3,22) = 4.680, p = 0.011. *	Log ratio comparisons:<br>VZ: p > 0.05. ns<br>IZ: p < 0.05. *<br>DL: p > 0.05. ns<br>UL: p > 0.05. ns
**e, S22**	One between-subjects factor: Species (2 levels). Multivariate DVs: UL, DL, IZ, VZ. Omnibus tests on isometric log ratio transformed data. Samples (MS/FTD): 5/13	No.<br>Henze-Zirkler (MS: HZ = 0.49784, p = 0.2449; FTD: HZ = 1.01, p = 0.00367). ** Box's M (Chi2(6) = 14.771, p = 0.0221). *	Npmv:<br>Wilks' Lambda<br>W(3,14) = 15.583, p < 0.001. ***	Log ratio comparisons.<br>VZ: p < 0.01. **<br>IZ: p < 0.05. *<br>DL: p < 0.01. **<br>UL: p > 0.05. ns
**e, S23**	One between-subjects factor: Species (2 levels). Multivariate DVs: UL, DL, IZ, VZ. Omnibus tests on isometric log ratio transformed data. Samples (MS/FTD): 9/3	No.<br>Henze-Zirkler (MS: HZ = 0.5723, p = 0.235, FTD: HZ = 12, p < 0.001). ***<br>Box's M: Chi2(6) = 51.064, p < 0.001. ***	Npmv:<br>ANOVA-type<br>F(2.474, 15.8322) = 25.287, p < 0.001. ***	Log ratio comparisons.<br>VZ: p < 0.01. **<br>IZ: p > 0.05. ns<br>DL: p < 0.05. *<br>UL: p < 0.01. **
**e, S24**	One between-subjects factor: Species (2 levels). Multivariate DVs: UL, DL, IZ, VZ. Omnibus tests on isometric log ratio transformed data. Samples (MS/FTD): 3/3	No.<br>Henze-Zirkler (HZ = 0.44, p = 0.4666). ns<br>Box's M (Chi2(6) = 24.767, p = 0.00038). **	Npmv:<br>Wilks' Lambda<br>W(1.535,6.185) = 5.207, p = 0.106. ns	Log ratio comparisons.<br>VZ: p > 0.05. ns<br>IZ: p > 0.05. ns<br>DL: p > 0.05. ns<br>UL: p > 0.05. ns
**e, S26**	One between-subjects factor: Species (2 levels). Multivariate DVs: UL, DL, IZ, VZ. Omnibus tests on isometric log ratio transformed data. Samples (MS/FTD): 3/3	No.<br>Henze-Zirkler (MS: HZ = 12, p < 0.001; FTD: HZ = 12, p < 0.001). ***<br>Box's M (Chi2(6) = 56.495, p < 0.0001). ***	Npmv:<br>ANOVA-type<br>F(2.176,8.7025) = 2.563, p = 0.131. ns	Log ratio comparisons.<br>VZ: p > 0.05. ns<br>IZ: p > 0.05. ns<br>DL: p > 0.05. ns<br>UL: p > 0.05. ns
**f, S24**	One between-subjects factor: Species (2 levels). Multivariate DVs: UL, DL, IZ, VZ. Omnibus tests on isometric log ratio transformed data. Samples (MS/FTD): 9/7	No.<br>Henze-Zirkler (MS: HZ = 36, p < 0.0001; FTD: HZ = 0.5342, p = 0.2528). ***<br>Box's M (Chi2(6) = 297.02, p < 0.0001). ***	Npmv:<br>Wilks' Lambda<br>W(3,12) = 19.934, p < 0.001. ***	Log ratio comparisons.<br>VZ: p < 0.01. **<br>IZ: p < 0.01. **<br>DL: p < 0.01. **<br>UL: p > 0.05. ns
**f, S25**	One between-subjects factor: Species (2 levels). Multivariate DVs: UL, DL, IZ, VZ. Omnibus tests on isometric log ratio transformed data. Samples (MS/FTD): 8/9	No.<br>Henze-Zirkler (MS: HZ = 0.837, p = 0.0123; FTD: HZ = 0.51144, p = 0.3843). *<br>Box's M (Chi2(6) = 38.385, p < 0.0001). ***	Npmv:<br>ANOVA-type<br>F(2.345, 35.0125) = 45.632, p < 0.001. ***	Log ratio comparisons.<br>VZ: p < 0.01. **<br>IZ: p < 0.01. **<br>DL: p < 0.01. **<br>UL: p < 0.01. **
**f, S26**	One between-subjects factor: Species (2 levels). Multivariate DVs: UL, DL, IZ, VZ. Omnibus tests on isometric log ratio transformed data. Samples (MS/FTD): 5/4	Yes.<br>Henze-Zirkler (MS: HZ = 0.4709, p = 0.31225; FTD: HZ = 0.4185, p = 0.3935). ns Box's M: Chi2(6) = 17.449, p = 0.00777. ns	MANOVA:<br>Wilks' Lambda W(3,5) = 11.0712, p = 0.012. *	Log ratio comparisons.<br>VZ: p < 0.01. **<br>IZ: p < 0.01. **<br>DL: p < 0.01. **<br>UL: p < 0.01. **
**f, S27**	One between-subjects factor: Species (2 levels). Multivariate DVs: UL, DL, IZ, VZ. Omnibus tests on isometric log ratio transformed data. Samples (MS/FTD): 4/5	Yes.<br>Henze-Zirkler (MS: HZ = 0.4185, p = 0.3934; FTD: HZ = 0.5248, p = 0.18913). ns<br>Box's M (Chi2(6) = 15.685, p = 0.01554). ns	MANOVA:<br>Wilks' Lambda W(3,5) = 18.1560, p = 0.004. **	Log ratio comparisons.<br>VZ: p < 0.01. **<br>IZ: p < 0.01. **<br>DL: p < 0.01. **<br>UL: p < 0.01. **
**f, S28**	One between-subjects factor: Species (2 levels). Multivariate DVs: UL, DL, IZ, VZ. Omnibus tests on isometric log ratio transformed data. Samples (MS/FTD): 4/7	No.<br>Henze-Zirkler (MS: HZ = 0.4185, p = 0.3934; FTD: HZ = 1.3132, p < 0.001). ***<br>Box's M (Chi2(6) = 40.90, p < 0.0001). ***	Npmv:<br>ANOVA-type<br>F(2.203, 17.6248) = 2.525, p = 0.105. ns	Log ratio comparisons.<br>VZ: p > 0.05. ns<br>IZ: p > 0.05. ns<br>DL: p > 0.05. ns<br>UL: p > 0.05. ns
**i**	One between-subjects factor (2 levels). Examining a ratio value (NEUROD1$^+$/TdTom$^+$)/(SOX2$^+$/TdTom$^+$). Samples (MS/FTD): 4/4	Yes.<br>Shapiro-Wilk (W = 0.938, p= 0.593).<br>Levene's test (W = 0.762, p=0.416). ns	NA	t-test.<br>t(6) = -2.665, p = 0.0373. *

**Figure 3**				
**Panel**	**Groups**	**Parametric**	**Omnibus**	**Post-hoc/Pairwise tests**
**a**	Two between-subjects factors: Species (2 levels), Days post EP (4 levels). Examining deep layer TdTom EP leading edge. Samples (MS/FTD):<br>Day1: 5/7<br>Day2: 5/8<br>Day3: 9/6<br>Day4: 3/8	No.<br>Shapiro-Wilk (W = 34, p = 0.8843). ns<br>Levene's test (F(7,43) = 4.5341, p < 0.001). ***	NA	Pairwise Mann-Whitney U tests:<br>Day1: U = 34.0, p = 0.0051. **<br>Day2: U = 40.0, p = 0.00155. **<br>Day3: U = 41.0, p = 0.1135. ns<br>Day4: U = 21.0, p = 0.0848. ns

**b**	Two between-subjects factors: Species (2 levels), Days post EP (4 levels). Examining upper layer TdTom EP leading edge. Samples (MS/FTD):<br>Day1: 9/3<br>Day3: 5/4<br>Day5: 4/3<br>Day7: 9/3	No.<br>Shapiro-Wilk ($W = 0.926$, $p = 0.01185$). *	NA	Pairwise Mann-Whitney U tests:<br>Day1: $U = 26.0$, $p = 0.0364$. *<br>Day3: $U = 20.0$, $p = 0.0364$. *<br>Day5: $U = 12.0$, $p = 0.0762$. ns<br>Day7: $U = 17.0$, $p = 0.6$. ns
**d, Day1**	One between-subjects factor: Species (2 levels). Multivariate DV: Above TBR2, TBR2 only, PAX6$^+$TBR2$^+$, PAX6 only. Omnibus tests on isometric log ratio transformed data. Samples (MS/FTD): 5/11.	No.<br>Henze-Zirkler (MS: $HZ = 0.51169$, $p = 0.2148$; FTD: $HZ = 1.24897$, $p = 0.00016$). **<br>Box's M (Chi2(6) = 34.8434, $p < 0.0001$). ***	Npmv:<br>Wilks' Lambda $W(3,12) = 29.521$, $p < 0.001$. ***	Log ratio comparisons:<br>PAX6 only: $p < 0.01$. **<br>PAX6$^+$TBR2$^+$: $p < 0.05$. *<br>TBR2 only: $p < 0.01$. **<br>Above TBR2: $p < 0.01$. **
**d, Day2**	One between-subjects factor: Species (2 levels). Multivariate DV: Above TBR2, TBR2 only, PAX6$^+$TBR2$^+$, PAX6 only. Omnibus tests on isometric log ratio transformed data. Samples (MS/FTD): 7/13	No.<br>Henze-Zirkler (MS: $HZ = 0.51172$, $p = 0.3068$. ns<br>FTD: $HZ = 0.64$, $p = 0.1896$). ns<br>Box's M (Chi2(6) = 27.334, $p = 0.000125$). **	Npmv:<br>Wilks' Lambda $W(3,16) = 47.455$, $p < 0.001$. ***	Log ratio comparisons:<br>PAX6 only: $p < 0.01$. **<br>PAX6$^+$TBR2$^+$: $p < 0.05$. *<br>TBR2 only: $p > 0.05$. ns<br>Above TBR2: $p < 0.01$. **
**d, Day3**	One between-subjects factor: Species (2 levels). Multivariate DV: Above TBR2, TBR2 only, PAX6$^+$TBR2$^+$, PAX6 only. Omnibus tests on isometric log ratio transformed data. Samples (MS/FTD): 16/13	No.<br>Henze-Zirkler (MS: $HZ = 0.4798$, $p = 0.6667$. ns<br>FTD: $HZ = 1.29562$, $p = 0.0001238$). **<br>Box's M (Chi2(6) = 73.754, $p < 0.0001$). ***	Npmv:<br>ANOVA-type $F(2.336,62.2865) = 10.045$, $p < 0.001$. ***	Log ratio comparisons:<br>PAX6 only: $p < 0.01$. **<br>PAX6$^+$TBR2$^+$: $p < 0.05$. *<br>TBR2 only: $p > 0.05$. ns<br>Above TBR2: $p < 0.01$. **

**Figure 4**				
**Panel**	**Groups**	**Parametric**	**Omnibus**	**Post-hoc/Pairwise tests**
**c**	One between-subjects factor: Condition (2 levels; CAG-Cre vs Emx2-Cre). Multivariate DV: VZ, IZ, CP. Omnibus tests on isometric log ratio transformed data. Samples (MS/FTD): 4/4.	Yes.<br>Henze-Zirkler (CAG-Cre: $HZ = 0.18663$, $p = 0.66586$. ns<br>Emx2-Cre: $HZ = 0.3465$, $p = 0.1748$). ns<br>Box's M (Chi2(3) = 12.98605, $p = 0.004667$). **	MANOVA:<br>Wilks' Lambda $W(2,5)=31.9837$, $p = 0.0014$. **	Log ratio comparisons:<br>VZ: $p < 0.01$. **<br>IZ: $p > 0.05$. ns<br>CP: $p < 0.01$. **
**f**	One between-subjects factor: Condition (2 levels; CAG-Cre vs Emx2-Cre). Multivariate DV: VZ, IZ, CP. Omnibus tests on isometric log ratio transformed data. Samples (MS/FTD): 4/4	Yes.<br>Henze-Zirkler (CAG-Cre: $HZ = 0.18459$; $p = 0.67465$. ns<br>Emx2-Cre: $HZ = 0.233$, $p = 0.47578$). ns<br>Box's M (Chi2(3) = 5.287365, $p = 0.15192$). ns	MANOVA:<br>Wilks' Lambda $W(2,5)=6.9548$, $p = 0.036$. *	Log ratio comparisons:<br>L6: $p < 0.01$. **<br>L5: $p < 0.05$. *<br>L2/4: $p < 0.01$. **
**i**	One between-subjects factor: Condition (2 levels). Examining the percentage of GFP$^+$ cells that are also SATB2$^+$. Samples (MS/FTD): 4/4.	Yes.<br>Shapiro-Wilk ($W = 0.917292$, $p = 0.408278$). ns	NA	*t*-test:<br>$t(6) = -4.6305$, $p = 0.003576$. **

**Figure 5**			
**Panel**	**Groups**	**Parametric**	**Pairwise tests**
**h, VZ**	Two between-subjects factors: Species (2 levels), Day (3 levels). Examining the density of PH3$^+$ cells in the ventricular zone.<br>Samples (MS/FTD):<br>Day1: 5/7<br>Day2: 6/7<br>Day3: 9/6	Yes.<br>Shapiro-Wilk ($W = 0.985$, $p = 0.859$). ns<br>Levene's test (Species; $W = 0.000273$), $p = 0.987$). ns<br>Levene's test (Day; $W = 1.978$, $p = 0.153$). ns	Pairwise *t*-tests:<br>Day1: $t(9.66) = -0.833$, $p = 0.818$. ns<br>Day2: $t(10.69) = -0.595$, $p = 0.818$. ns<br>Day3: $t(12.55) = 0.235$, $p = 0.818$. ns
**h, SVZ**	Two between-subjects factors: Species (2 levels), Day (3 levels). Examining the density of PH3$^+$ cells in the subventricular zone.<br>Samples (MS/FTD):<br>Day1: 5/7<br>Day2: 6/7<br>Day3: 9/6	No.<br>Shapiro-Wilk ($W = 1.978$, $p = 0.153$). ns<br>Levene's test (Species; $W = 7.469$, $p = 0.0095$). **<br>Levene's test (Day; $W = 4.049$, $p = 0.0257$). *	Pairwise Mann-Whitney *U* tests:<br>Day1: $U = 33$, $p = 0.01416$. *<br>Day2: $U = 40$, $p = 0.00816$. **<br>Day3: $U = 51$, $p = 0.00816$. **
**i, VZ**	Two between-subjects factors: Species (2 levels), Day (3 levels). Examining the proportion of TdTom$^+$PH3$^+$/TdTom$^+$ in VZ.<br>Samples (MS/FTD):<br>Day1: 5/7<br>Day2: 4/7<br>Day3: 9/6	Yes.<br>Shapiro-Wilk ($W = 0.976$, $p = 0.584$). ns<br>Levene's test (Species; $W = 0.259$, $p = 0.6136$). ns<br>Levene's test (Day; $W = 0.1532$, $p = 0.859$). ns	Pairwise *t*-tests:<br>Day1: $t(4.55)= 1.353$, $p = 0.279$. ns<br>Day2: $t(7.51) = 1.67$, $p = 0.279$. ns<br>Day3: $t(12.27) = 1.98$, $p = 0.2128$. ns
**i, SVZ**	Two between-subjects factors: Species (2 levels), Day (3 levels). Examining the proportion of TdTom$^+$PH3$^+$/TdTom$^+$ in SVZ.<br>Samples (MS/FTD):<br>Day1: 5/7<br>Day2: 4/7<br>Day3: 9/6	No.<br>Shapiro-Wilk ($W = 0.6459$, $p < 0.0001$). ***<br>Levene's test ($W = 9.026$, $p = 0.00482$). **	Pairwise Mann-Whitney *U* tests:<br>Day1: $U = 35.0$, $p = 0.004691$. **<br>Day2: $U = 28.0$, $p = 0.004691$. **<br>Day3: $U = 45.0$, $p = 0.019981$. *
**l**	One between-subjects factor: Species (2 levels). Examining the ratio of PH3$^+$TBR2$^+$ cells over all TBR2$^+$ cells. Samples (MS/FTD): 6/8.	No.<br>Shapiro-Wilk ($W = 0.734$, $p = 0.000849$). **<br>Levene's test ($W = 6.725$, $p = 0.0235$). *	Mann Whitney *U* test:<br>$U = 44.0$, $p = 0.003348$. **

**o**	One between-subjects factor: Species (2 levels). Examining the ratio of TBR2$^+$PCNA$^+$ cells over all TBR2$^+$ cells. Samples (MS/FTD): 5/5	Yes.<br>Shapiro-Wilk (W = 0.9228, p = 0.38051). ns<br>Levene's test (W = 4.629, p = 0.06362). ns	t-test.<br>$t(8)$ = 10.0987, p < 0.0001. (0.000008). ***
**r**	One between-subjects factor: Species (2 levels). Examining the ratio of PCNA$^+$SOX2$^+$ cells over all SOX2$^+$ cells. Samples (MS/FTD): 5/5	No.<br>Shapiro-Wilk (W = 0.79, p = 0.0111). *<br>Levene's test (W = 2.374, p = 0.16194). ns	Mann Whitney U test:<br>U = 0.0, p = 0.007937. **

**Figure 6**			
**Panel**	**Groups**	**Parametric**	**Pairwise tests**
**c, S20-21**	One between-subjects factor: Position (2 levels). Examining the length of initial axons. Samples (MS Medial/MS Lateral): 5/5	No.<br>Shapiro-Wilk (W = 0.832, p = 0.03521). *	Mann-Whitney U test:<br>U = 7.5, p = 0.1797. ns
**c, S20-22**	One between-subjects factor: Position (2 levels). Examining the length of initial axons. Samples (MS Medial/MS Lateral): 6/6	No.<br>Shapiro-Wilk (W = 0.8186, p = 0.01536). *	Mann-Whitney U test:<br>U = 4, p = 0.02597. *
**f, S20-21**	One between-subjects factor (2 levels). Examining the length of initial axons. Samples: (FTD Medial/ FTD Lateral): 21/21	No.<br>Shapiro-Wilk (W = 0.8722, p = 0.000235) ***	Mann-Whitney U test:<br>U = 3.0, p < 0.0001. ***
**f, S20-22**	One between-subjects factor (2 levels). Examining the length of initial axons. Samples: (FTD Medial/ FTD Lateral): 7/7	Yes.<br>Shapiro-Wilk (W = 0.93182, p = 0.32355). ns	t-test:<br>$t(12)$ = 28.066, p < 0.0001. ***
**g**	One between-subjects factor: Position (2 levels). Examining the length of the axon bundle. FTD samples: 6	Yes.<br>Shapiro-Wilk (W = 0.9024, p = 0.38813). ns	Paired t-test:<br>$t(5)$ = -4.428, p = 0.00684. **
**i**	Continuous IV: EP angle (degrees); examining the length of the longest axon.<br>Samples: 14	NA	Linear regression:<br>p = 0.03. *<br>r = 0.579.
**l**	One between-subjects factor (2 levels). Examining the distance to crossing the midline. Samples (FTD neocortex injection/FTD cingulate cortex injection): 3/3	Yes.<br>Shapiro-Wilk (W = 0.963256, p = 0.844453). ns	t-test:<br>$t(4)$ = 12.211, p = 0.000258. ***

**Figure 7**			
**Panel**	**Groups**	**Parametric**	**Pairwise tests**
**f**	Comparison between species (2 species) at each stage (5 stages). Examining length of medial, deep layer longest axon as percentage of cortical width.<br>Samples (MS/FTD):<br>S21: 5/21<br>S22: 6/7<br>S23: 10/3<br>S24: 3/4<br>S26: 6/6	No.<br>Shapiro-Wilk.<br>W = 0.73595<br>p < 0.0001. ***	Pairwise Mann-Whitney U tests:<br>S21: U =37.5, p = 0.25177. ns<br>S22: U = 0, p = 0.005411. **<br>S23: U = 2.0, p = 0.04662. *<br>S24: U = 5.0, p = 0.57143. ns<br>S26: U =36, p = 0.005411. **
**g**	Comparison between species (2 species) at each stage (4 stages). Examining length of lateral, deep layer longest axon as percentage of cortical width.<br>Samples (MS/FTD):<br>S21: 5/21<br>S22: 6/7<br>S23: 10/3<br>S24: 3/4	No.<br>Shapiro-Wilk<br>W = 0.911, p = 0.000398. ***	Pairwise Mann-Whitney U tests:<br>S21: U = 3.0, p = 0.00284. **<br>S22: U = 0.0, p = 0.00284. **<br>S23: U = 0.0, p = 0.01726. *<br>S24: U = 0.0, p = 0.057143. ns
**h**	Comparison between species (2 species) at each stage (5 stages). Examining length of commissural, deep layer longest axon as percentage of cortical width.<br>Samples (MS/FTD):<br>S21: 5/21<br>S22: 6/7<br>S23: 10/3<br>S24: 3/4<br>S26: 6/6	No.<br>Shapiro-Wilk<br>W = 0.892537, p < 0.0001. ***	Pairwise Mann-Whitney U tests:<br>S21: U = 0, p = 0.0029. **<br>S22: U = 0, p = 0.0029. **<br>S23: U = 0, p = 0.00874. **<br>S24: U = 0, p = 0.05714. ns<br>S26: U = 0, p = 0.0036. **
**i**	Comparison between species (2 species) at each stage (5 stages). Examining length of medial, upper layer longest axon as percentage of cortical width.<br>Samples (MS/FTD):<br>S24: 9/8<br>S25: 7/13<br>S26: 5/4<br>S27: 5/6<br>S28: 5/5	No.<br>Shapiro-Wilk (W = 0.57133, p < 0.0001. ***	Pairwise Mann-Whitney U tests:<br>S24: U =36.0, p = 1.0. ns<br>S25: U = 91.0, p = 0.000143. ***<br>S26: U = 0.0, p = 0.026455. *<br>S27: U = 4.0, p = 0.064935. ns<br>S28: U = 25.0, p = 0.019841. *
**j**	Comparison between species (2 species) at each stage (5 stages). Examining length of commissural, upper layer longest axon as percentage of cortical width.<br>Samples (MS/FTD):<br>S24: 9/8<br>S25: 7/13<br>S26: 5/4<br>S27: 5/6<br>S28: 5/5	No.<br>Shapiro-Wilk (W = 0.8785, p < 0.0001. ***	Pairwise Mann-Whitney U tests:<br>S24: U = 0, p = 0.000542. ***<br>S25: U = 0, p = 0.000129. ***<br>S26: U = 0, p = 0.015873. *<br>S27: U = 0, p = 0.007215. **<br>S28: U = 0, p = 0.009921. **
**k**	Comparison between species (2 species) at each day post electroporation (4 days). Examining length of medial, deep layer longest axon as percentage of cortical width. Samples (MS/FTD):<br>Day1: 5/7<br>Day2: 6/7<br>Day3: 10/10<br>Day4: 3/4	No.<br>Shapiro-Wilk (W = 0.777, p < 0.001). ***	Pairwise Mann-Whitney U tests:<br>Day1: U = 17.5, p = 1.9. ns<br>Day2: U = 38.5, p = 0.01125. *<br>Day3: U = 100, p = 0.00035. ***<br>Day4: U = 12, p = 0.04255. *

**l**	Comparison between species (2 species) at each day post electroporation (4 days). Examining length of lateral, deep layer longest axon as percentage of cortical width. Samples (MS/FTD):<br>Day1: 5/7<br>Day2: 6/7<br>Day3: 10/10<br>Day4: 3/4	No.<br>Shapiro-Wilk ($W = 0.9362$, $p = 0.00788$). **	Pairwise Mann-Whitney U tests:<br>Day1: $U = 24.5$, $p = 0.138971$. ns<br>Day2: $U = 38$, $p = 0.073$. ns<br>Day3: $U = 30$, $p = 0.138971$. ns<br>Day4: $U = 0$, $p = 0.1143$. ns
**m**	Comparison between species (2 species) at each day post electroporation (4 days). Examining length of commissural, deep layer longest axon as percentage of cortical width. Samples (MS/FTD):<br>Day1: 5/7<br>Day2: 6/7<br>Day3: 10/10<br>Day4: 3/4	No.<br>Shapiro-Wilk ($W = 0.8654$, $p < 0.0001$). ***	Pairwise Mann-Whitney U tests:<br>Day1: $U = 17.5$, $p = 1.0$. ns<br>Day2: $U = 23$, $p = 1.0$. ns<br>Day3: $U = 65$, $p = 1.0$. ns<br>Day4: $U = 8$, $p = 1.0$. ns
**n**	Comparison between species (2 species) at each day post electroporation (4 days). Examining length of medial, upper layer longest axon as percentage of cortical width. Samples (MS/FTD):<br>Day1: 9/3<br>Day3: 5/5<br>Day5: 5/4<br>Day7: 9/6	No.<br>Shapiro-Wilk ($W = 0.58$, $p < 0.0001$). ***	Pairwise Mann-Whitney U tests:<br>Day1: $U = 13.5$, $p = 1.0$. ns<br>Day3: $U = 25.0$, $p = 0.0149$. *<br>Day5: $U = 20$, $p = 0.00201$. **<br>Day7: $U = 54$, $p = 0.005031$. **
**o**	Comparison between species (2 species) at each day post electroporation (4 days). Examining length of commissural, upper layer longest axon as percentage of cortical width. Samples (MS/FTD):<br>Day1: 9/3<br>Day3: 5/5<br>Day5: 5/4<br>Day7: 9/6	No.<br>Shapiro-Wilk ($W = 0.69276$, $p < 0.0001$). ***	Pairwise Mann-Whitney U tests:<br>Day1: $U = 13.5$, $p = 1.0$. ns<br>Day3: $U = 0.0$, $p = 0.015873$. *<br>Day5: $U = 2.0$, $p = 0.148148$. ns<br>Day7: $U = 0.0$, $p = 0.001598$. **

**Figure 8**				
**Panel**	**Groups**	**Parametric**	**Omnibus**	**Pairwise tests**
**d**	Comparison between upper and deep layer EP. Middle 20% of values at each layer (7 levels).<br>Samples (DL/UL): 3/3	Yes.<br>Shapiro-Wilk ($W = 0.97$, $p = 0.3356$). ns	Two-way ANOVA. No main effect of EP ($F(1,28) = 2.582$, $p = 0.119$). ns<br>No main effect of Layer ($F(6,28) = 1.688$, $p = 0.161$). ns<br>No EP*Layer interaction ($F(6,28) = 0.638$, $p = 0.699$). ns	NA
**f**	Comparison between upper and deep layer EP. Middle 20% of values at each layer (7 levels).<br>Samples (DL/UL): 7/5	Yes.<br>Shapiro-Wilk ($W = 0.981$, $p = 0.268$). ns	Two-way ANOVA. Main effect of EP ($F(1,70) = 14.37$, $p = 0.000315$). ***<br>Main effect of Layer ($F(6,70) = 5.2774$, $p = 0.000155$). ***<br>EP*Layer effect ($F(6,70) = 4.3616$, $p = 0.00085$). ***	Pairwise t-tests:<br>L1: $t(9.475) = 1.010$, $p = 0.591$. ns<br>L2/3: $t(9.588) = 0.589$, $p = 0.778$. ns<br>L4: $t(8.314) = 0.373$, $p = 0.778$. ns<br>L5: $t(9.760) = 1.252$, $p = 0.559$. ns<br>L6: $t(6.642) = 2.292$, $p = 0.202$. ns<br>WM: $t(7.302) = 4.641$, $p = 0.015$. *<br>VZ: $t(7.390) = -0.293$, $p = 0.778$. ns
**h**	Comparison between upper and deep layer EP. Middle 20% of values at each layer (7 levels).<br>Samples (DL/UL): 3/3	Yes.<br>Shapiro-Wilk ($W = 0.98$, $p = 0.663$). ns	Two-way ANOVA. No main effect of EP ($F(1,28) = 0.053$, $p = 0.819$). ns<br>No main effect of Layer ($F(6,28) = 1.495$, $p = 0.216$). ns<br>No EP*Layer interaction ($F(6,28) = 0.0396$, $p = 0.999$). ns	NA
**j**	Comparison between upper and deep layer EP. Middle 20% of values at each layer (7 levels).<br>Samples (DL/UL): 3/6	No.<br>Shapiro-Wilk ($W = 0.86$, $p < 0.0001$). ***	Aligned Rank Transform. No main effect of EP ($F(1,49) = 0.387$, $p = 0.5369$). ns<br>Main effect of Layer ($F(6,49) = 8.596$, $p < 0.0001$).***<br>No EP*Layer interaction ($F(6,49) = 1.075$), $p = 0.3906$. ns	NA

**Supplementary Figure 2**			
**Panels**	**Groups**	**Parametric**	**Pairwise tests**
**a**	Two between-subjects factors: Species (2 levels), Stage (5 levels). Cortical width following deep layer EP. Subjects (MS/FTD):<br>S21: 5/21<br>S22: 5/13<br>S23: 9/3<br>S24: 3/3<br>S26: 3/3	Yes.<br>Shapiro-Wilk ($W = 0.988$, $p = 0.747$). ns<br>Levene's test ($W = 1.1088$, $p = 0.3713$). ns	Pairwise t-tests.<br>S21: $t(7.998) = -1.088$, $p = 0.308$. ns<br>S22: $t(8.668) = 3.417$, $p = 0.020$. *<br>S23: $t(2.348) = 3.223$, $p = 0.085$. ns<br>S24: $t(4) = 11.870$, $p = 0.001$. **<br>S26: $t(4) = 3.355$, $p = 0.047$. *
**b**	Two between-subjects factors: Species (2 levels), Stage (5 levels). Leading edge from the VZ after DL EP. Subjects (MS/FTD):<br>S21: 5/21<br>S22: 5/13<br>S23: 9/3<br>S24: 3/3<br>S26: 3/3	Yes.<br>Shapiro-Wilk ($W = 0.977$, $p = 0.229$). ns<br>Levene's test ($W = 2.054$, $p = 0.049$). *	Pairwise t-tests.<br>S21: $t(15.986) = -4.023$, $p = 0.002$. **<br>S22: $t(12.527) = 2.873$, $p = 0.022$. *<br>S23: $t(2.405) = 3.021$, $p = 0.075$. ns<br>S24: $t(4) = 15.817$, $p < 0.0001$. ***<br>S26: $t(4) = 3.371$, $p = 0.035$. *
**c**	Two between-subjects factors: Species (2 levels), Stage (5 levels). Mean position of most advanced 2% of cells after DL EP.	No.	Pairwise Mann-Whitney U tests.<br>S21: $U = 99.0$, $p = 0.004$. **

	S21: 5/21<br>S22: 5/13<br>S23: 9/3<br>S24: 3/3<br>S26: 3/3	Shapiro-Wilk ($W = 0.839$, $p < 0.0001$). ***<br>Levene's test ($W = 0.940$, $p = 0.498$). ns	S22: $U = 54.0$, $p = 0.088$. ns<br>S23: $U = 19.0$, $p = 0.373$. ns<br>S24: $U = 0.0$, $p = 0.167$. ns<br>S26: $U = 1.0$, $p = 0.250$. ns
**d**	Two between-subjects factors: Species (2 levels), Stage (5 levels). Cortical width following upper layer EP. Subjects (MS/FTD):<br>S24: 9/7<br>S25: 8/9<br>S26: 5/4<br>S27: 4/5<br>S28: 4/7	Yes.<br>Shapiro-Wilk ($W = 0.985$, $p = 0.626$). ns<br>Levene's test ($W = 1.605$, $p = 0.138$). ns	Pairwise *t*-tests.<br>S24: $t(12.572) = 13.620$, $p < 0.0001$. ***<br>S25: $t(11.607) = 11.357$, $p < 0.0001$. ***<br>S26: $t(6.011) = 6.335$, $p = 0.001$. **<br>S27: $t(5.246) = 3.462$, $p = 0.017$. *<br>S28: $t(4.808) = 5.685$, $p = 0.003$. **
**e**	Two between-subjects factors: Species (2 levels), Stage (5 levels). Leading edge from the VZ after upper layer EP. Subjects (MS/FTD):<br>S24: 9/7<br>S25: 8/9<br>S26: 5/4<br>S27: 4/5<br>S28: 4/7	Yes.<br>Shapiro-Wilk ($W = 0.985$, $p = 0.643$). ns<br>Levene's test ($W = 1.569$, $p = 0.149$). ns	Pairwise *t*-tests.<br>S24: $t(7.397) = 0.015$, $p = 0.988$. ns<br>S25: $t(12.454) = -4.375$, $p = 0.004$. **<br>S26: $t(4.911) = 1.993$, $p = 0.13$. ns<br>S27: $t(6.879) = 3.951$, $p = 0.014$. *<br>S28: $t(3.702) = 4.5$, $p = 0.022$. *
**f**	Two between-subjects factors: Species (2 levels), Stage (5 levels). Mean position of most advanced 2% of cells after UL EP.<br>S24: 9/7<br>S25: 8/9<br>S26: 5/4<br>S27: 4/5<br>S28: 4/7	No.<br>Shapiro-Wilk ($W = 0.941$,$p = 0.005$). **<br>Levene's test ($W = 4.798$, $p = 0.00011$). **	Pairwise Mann-Whitney *U* tests.<br>S24: $U = 63.0$, $p < 0.0001$. ***<br>S25: $U = 72.0$, $p < 0.0001$. ***<br>S26: $U = 18.0$, $p = 0.079$. ns<br>S27: $U = 1.0$, $p = 0.053$. ns<br>S28: $U = 7.0$, $p = 0.230$. ns

**Supplementary Figure 3**				
**Panels**	**Groups**	**Parametric**	**Omnibus**	**Posthoc/Pairwise tests**
**a**	Two between-subjects factors: Species (2 levels), Day post EP (4 levels). Cortical width after deep layer EP (MS/FTD).<br>Day1: 5/7<br>Day2: 5/8<br>Day3: 9/6<br>Day4: 3/8	Yes.<br>Shapiro-Wilk ($W = 0.979$, $p = 0.484$). ns<br>Levene's test ($W = 0.899$, $p = 0.516$). ns	NA	Pairwise *t*-tests.<br>Day1: $t(9.191) = 2.992$, $p = 0.015$. *<br>Day2: $t(5.325) = 10.274$, $p < 0.0001$. ***<br>Day3: $t(10.45) = 10.555$, $p < 0.0001$. ***<br>Day4: $t(5.188) = 14.043$, $p < 0.0001$. ***
**b**	Two between-subjects factors: Species (2 levels), Day post EP (4 levels). The leading edge from the ventricular zone after deep layer EP (MS/FTD).<br>Day1: 5/7<br>Day2: 5/8<br>Day3: 9/6<br>Day4: 3/8	No.<br>Shapiro-Wilk ($W = 0.974$, $p = 0.309$). ns<br>Levene's test ($W = 3.603$, $p = 0.004$). **	NA	Pairwise Mann-Whitney *U* tests:<br>Day1: $U = 35$, $p = 0.003$. **<br>Day2: $U = 40$, $p = 0.003$. **<br>Day3: $U = 54$, $p = 0.002$. **<br>Day4: $U = 24$, $p = 0.012$. *
**c**	Two between-subjects factors: Species (2 levels), Day post EP (4 levels). Cortical width after upper layer EP. Subjects (MS/FTD):<br>Day1: 9/3<br>Day3: 5/4<br>Day5: 4/3<br>Day7: 9/3	No.<br>Shapiro-Wilk ($W = 0.969$, $p = 0.346$). ns<br>Levene's test ($W = 2.904$, $p = 0.018$). *	NA	Pairwise Mann-Whitney *U* tests:<br>Day1: $U = 27$, $p = 0.018$. *.<br>Day3: $U = 20$, $p = 0.021$. *<br>Day5: $U = 12$, $p = 0.057$. ns<br>Day7: $U = 27$, $p = 0.018$. *
**d**	Two between-subjects factors: Species (2 levels), Day post EP (4 levels). Cortical width after upper layer EP. Subjects (MS/FTD):<br>Day1: 9/3<br>Day3: 5/4<br>Day5: 4/3<br>Day7: 9/3	No.<br>Shapiro-Wilk ($W = 0.943$, $p = 0.042$). *<br>Levene's test ($W = 3.139$, $p = 0.012$). *	NA	Pairwise Mann-Whitney *U* tests:<br>Day1: $U = 27$, $p = 0.018$. *.<br>Day3: $U = 20$, $p = 0.021$. *<br>Day5: $U = 12$, $p = 0.057$. ns<br>Day7: $U = 27$, $p = 0.018$. *
**o, Day1**	One between-subjects factor: Species (2 levels).<br>Multivariate DV. Omnibus tests on isometric log ratio transformed data. Subjects (MS/FTD): 6/9	No.<br>Henze-Zirkler ($HZ = 1.388$, $p < 0.0001$). ***<br>Box's M (Chi2(10) = 212.855, $p < 0.0001$). ***	Npmv:<br>Wilks' Lambda ($W(4,10) = 24.675$, $p < 0.0001$). ***	Log ratio comparisons.<br>Under TBR2: $p < 0.01$. **<br>In TBR2: $p < 0.01$. **<br>In faint TBR1: $p < 0.01$. **<br>In strong TBR1: $p > 0.05$. ns<br>Above strong TBR1: $p > 0.05$. ns
**o, Day2**	One between-subjects factor: Species (2 levels).<br>Multivariate DV. Omnibus tests on isometric log ratio transformed data. Subjects (MS/FTD): 7/12	No.<br>Henze-Zirkler ($HZ = 1.440$, $p < 0.0001$). ***<br>Box's M (Chi2(10) = 46.182, $p < 0.0001$). ***	Npmv:<br>ANOVA-type ($F(3.152,48.954) = 33.656$, $p < 0.0001$). ***	Log ratio comparisons.<br>Under TBR2: $p < 0.01$. **<br>In TBR2: $p > 0.05$. ns<br>In faint TBR1: $p < 0.01$. **<br>In strong TBR1: $p < 0.01$. **<br>Above strong TBR1: $p > 0.05$. ns
**o, Day3**	One between-subjects factor: Species (2 levels).<br>Multivariate DV. Omnibus tests on isometric log ratio transformed data. Subjects (MS/FTD): 13/8	No.<br>Henze-Zirkler ($HZ = 1.496$, $p < 0.0001$). ***<br>Box's M (Chi2(10) = 68.667, $p < 0.0001$). ***	Npmv:<br>Wilk's Lambda. ($W(2.631, 46.5322) = 17.578$, $p < 0.0001$). ***	Log ratio comparisons.<br>Under TBR2: $p < 0.01$. **<br>In TBR2: $p > 0.05$. ns<br>In faint TBR1: $p < 0.05$. *<br>In strong TBR1: $p > 0.05$. ns<br>Above strong TBR1: $p < 0.01$. **

Supplementary Figure 4				
**Panels**	**Groups**	**Parametric**	**Omnibus**	**Post-hoc/Pairwise tests**
**a**	One between-subjects factor: Species (2 levels). Multivariate DV. Omnibus tests on isometric log ratio transformed data.	Yes.<br>Henze-Zirkler (HZ = 0.1438, p = 0.9475). ns<br>Box's M (Chi2(3) = 7.542, p = 0.0565). ns	MANOVA.<br>Wilks' Lambda.<br>F(2, 5) = 0.0447, p = 0.9566. ns	Log ratio comparisons.<br>VZ: $p > 0.05$. ns<br>IZ: $p > 0.05$. ns<br>CP: $p > 0.05$. ns
**b**	One between-subjects factor: Species (2 levels). Multivariate DV. Omnibus tests on isometric log ratio transformed data.	Yes.<br>Henze-Zirkler (HZ = 0.37, p = 0.287). ns<br>Box's M (Chi2(3) = 1.574, p = 0.665). ns	MANOVA.<br>Wilks' Lambda W(2,5) = 0.662, p = 0.556. ns	Log ratio comparisons.<br>L2-4: $p > 0.05$. ns<br>L5: $p > 0.05$. ns<br>L6: $p > 0.05$. ns

Supplementary Figure 5			
**Panels**	**Groups**	**Parametric**	**Pairwise tests**
**d**	One within-subjects factor: EP (2 levels). Number of $PH3^+$ cells above the VZ. Samples (FTD): 6	Yes.<br>Shapiro-Wilk (W = 0.882, p = 0.2775). ns	Paired samples t-test (t(5) = 12.89, p < 0.0001 (p = 0.00005)). ***
**e**	One within-subjects factor: EP (2 levels). Number of $PH3^+$ cells above the VZ. Samples (FTD): 9	No.<br>Shapiro-Wilk (W = 0.828, p = 0.04278). *	Wilcoxon signed-rank test (W = 0.0, p = 0.0039). **
**f**	One within-subjects factor: EP (2 levels). Number of $PH3^+$ cells above the VZ. Samples (FTD): 6	Yes.<br>Shapiro-Wilk (W = 0.812, p = 0.0753). ns	Paired samples t-test (t(5) = 0.0, p = 1.0). ns
**g**	One within-subjects factor: EP (2 levels). Number of $PH3^+$ cells above the VZ. Samples (FTD): 5	No.<br>Shapiro-Wilk (W = 0.5522, p = 0.000131). ***	Wilcoxon signed-rank test (W = 0, p = 1.0). ns

Supplementary Figure 6			
**Panels**	**Groups**	**Parametric**	**Pairwise tests**
**c**	Two between-subjects factors: Species (2 levels), Stage (7 levels). Density of UL cingulate EdU^+ cells. Samples (MS/FTD):<br>S21: 4/4<br>S22: 4/3<br>S23: 4/4<br>S24: 4/4<br>S25: 4/3	Yes.<br>Shapiro-Wilk (W = 0.97, p = 0.38). ns<br>Levene's test (W = 0.717, p = 0.69). ns	Pairwise t-tests.<br>S21: t(6) = 0.731, p = 0.573. ns<br>S22: t(4.457) = 1.961, p = 0.285. ns<br>S23: t(6) = 1.105, p = 0.519. ns<br>S24: t(6) = -4.652, p = 0.017. *<br>S25: t(4.706) = 0.605, p = 0.573. ns

Supplementary Figure 7			
**Panels**	**Groups**	**Parametric**	**Pairwise tests**
**a**	Comparison between species (2 species) at each stage (5 stages). Examining length of medial, deep layer longest axon. Samples (MS/FTD):<br>S21: 5/21<br>S22: 6/7<br>S23: 10/3<br>S24: 3/4<br>S26: 6/6	No.<br>Shapiro-Wilk (W = 0.614 < 0.0001). ***<br>Levene's test (W = 15.386, p < 0.0001). ***	Pairwise Mann-Whitney U tests:<br>S21: U = 37.5, p = 0.252. ns<br>S22: U = 0.0, p = 0.005. **<br>S23: U = 16.0, p = 0.937. ns<br>S24: U = 12.0, p = 0.095. ns<br>S26: U = 36.0, p = 0.005. **
**b**	Comparison between species (2 species) at each stage (4 stages). Examining length of lateral, deep layer longest axon. Samples (MS/FTD):<br>S21: 5/21<br>S22: 6/7<br>S23: 10/3<br>S24: 3/4	No.<br>Shapiro-Wilk (W = 0.88, p < 0.0001). ***<br>Levene's test (W = 6.374, p < 0.0001). ***	Pairwise Mann-Whitney U tests:<br>S21: U = 3.0, p = 0.003. **<br>S22: U = 0.0, p = 0.003. **<br>S23: U = 0.0, p = 0.017. *<br>S24: U = 0.0, p = 0.057. ns
**c**	Comparison between species (2 species) at each stage (5 stages). Examining length of commissural, deep layer longest axon. Samples (MS/FTD):<br>S21: 5/21<br>S22: 6/7<br>S23: 10/3<br>S24: 3/4<br>S26: 6/6	No.<br>Shapiro-Wilk (W = 0.872, p < 0.0001). ***<br>Levene's test (W = 7.411, p < 0.0001). ***	Pairwise Mann-Whitney U tests:<br>S21: U = 0.0, p = 0.003. **<br>S22: U = 0.0, p = 0.003. **<br>S23: U = 0.0, p = 0.009. **<br>S24: U = 0.0, p = 0.057. ns<br>S26: U = 0.0, p = 0.004. **
**d**	Comparison between species (2 species) at each stage (5 stages). Examining length of medial, upper layer longest axon. Samples (MS/FTD):<br>S24: 9/8<br>S25: 7/13<br>S26: 5/4<br>S27: 5/6<br>S28: 5/5	No.<br>Shapiro-Wilk (W = 0.687, p < 0.0001). ***<br>Levene's test (W = 9.266, p < 0.0001). ***	Pairwise Mann-Whitney U tests:<br>S24: U = 36, p = 1.0. ns<br>S25: U = 91, p < 0.0001. ***<br>S26: U = 2, p = 0.079. ns<br>S27: U = 4, p = 0.079. ns<br>S28: U = 25, p = 0.02. *
**e**	Comparison between species (2 species) at each stage (5 stages). Examining length of commissural, upper layer longest axon. Samples (MS/FTD):<br>S24: 9/8<br>S25: 7/13<br>S26: 5/4<br>S27: 5/6<br>S28: 5/5	No.<br>Shapiro-Wilk (W = 0.884, p < 0.0001). ***<br>Levene's test (W = 15.99, p = 0.000163). **	Pairwise Mann-Whitney U tests:<br>S24: U = 0.0, p = 0.001. **<br>S25: U = 0.0, p < 0.0001. ***<br>S26: U = 0.0, p = 0.016. *<br>S27: U = 0.0, p = 0.007. **<br>S28: U = 0.0, p = 0.01. *
**f**	Comparison between species (2 species) at each day post electroporation (4 days). Examining length of medial, deep layer longest axon. Samples (MS/FTD):<br>Day1: 5/7<br>Day2: 6/7<br>Day3: 10/10<br>Day4: 3/4	No.<br>Shapiro-Wilk (W = 0.695, p < 0.0001). ***<br>Levene's test (W = 6.295, p < 0.0001). ***	Pairwise Mann-Whitney U tests:<br>Day1: U = 17.5, p = 1.0. ns<br>Day2: U = 38.5, p = 0.011. *<br>Day3: U = 100.0, p < 0.0001. ***<br>Day4: U = 12.0, p = 0.043. *

**g**	Comparison between species (2 species) at each day post electroporation (4 days). Examining length of lateral, deep layer longest axon. Samples (MS/FTD):<br>Day1: 5/7<br>Day2: 6/7<br>Day3: 10/10<br>Day4: 3/4	No.<br>Shapiro-Wilk ($W = 0.955$, $p = 0.046$). *<br>Levene's test ($W = 3.386$, $p = 0.0056$). **	Pairwise Mann-Whitney U tests:<br>Day1: $U = 24.5$, $p = 0.209$. ns<br>Day2: $U = 40$, $p = 0.033$. *<br>Day3: $U = 43$, $p = 0.829$. ns<br>Day4: $U = 6$, $p = 1.0$. ns
**h**	Comparison between species (2 species) at each day post electroporation (4 days). Examining length of commissural, deep layer longest axon. Samples (MS/FTD):<br>Day1: 5/7<br>Day2: 6/7<br>Day3: 10/10<br>Day4: 3/4	No.<br>Shapiro-Wilk ($W = 0.914$, $p = 0.001$). **<br>Levene's test ($W = 2.839$, $p = 0.0156$). *	Pairwise Mann-Whitney U tests:<br>Day1: $U = 17.5$, $p = 1.0$. ns<br>Day2: $U = 26.0$, $p = 0.691$. ns<br>Day3: $U = 85.0$, $p = 0.036$. *<br>Day4: $U = 12.0$, $p = 0.114$. ns
**i**	Comparison between species (2 species) at each day post electroporation (4 days). Examining length of medial, upper layer longest axon. Samples (MS/FTD):<br>Day1: 9/3<br>Day3: 5/5<br>Day5: 5/4<br>Day7: 9/6	No.<br>Shapiro-Wilk ($W = 0.654$, $p < 0.0001$). ***<br>Levene's test ($W = 5.42$, $p = 0.00023$). **	Pairwise Mann-Whitney U tests:<br>Day1: $U = 13.5$, $p = 1.0$. ns<br>Day3: $U = 25.0$, $p = 0.015$. *<br>Day5: $U = 20.0$, $p = 0.02$. *<br>Day7: $U = 54.0$, $p = 0.005$. **
**j**	Comparison between species (2 species) at each day post electroporation (4 days). Examining length of commissural, upper layer longest axon. Samples (MS/FTD):<br>Day1: 9/3<br>Day3: 5/5<br>Day5: 5/4<br>Day7: 9/6	No.<br>Shapiro-Wilk ($W = 0.809$, $p < 0.0001$). ***<br>Levene's test ($W = 4.611$, $p = 0.00083$). ***	Pairwise Mann-Whitney U tests:<br>Day1: $U = 13.5$, $p = 1.0$. ns<br>Day3: $U = 6.0$, $p = 0.296$. ns<br>Day5: $U = 20.0$, $p = 0.063$. ns<br>Day7: $U = 39.0$, $p = 0.296$. ns

Supplementary Table 3. Statistical tests used in manuscript and test outcomes: All comparisons were performed using the Python and R statistical analysis packages for statistical computing.
CP cortical plate; DL, deep layers; EP electroporation; FTD, fat-tailed dunnart; IZ, intermediate zone; L, layer; MS, mouse; MZ, marginal zone; ns, non-significant; S, stage; UL, upper layers; VZ ventricular zone.

SUPPLEMENTARY REFERENCES

1. Suárez, R. *et al.* Development of body, head and brain features in the Australian fat-tailed dunnart (Sminthopsis crassicaudata; Marsupialia: Dasyuridae); A postnatal model of forebrain formation. *PLOS ONE* **12**, e0184450 (2017).
2. Suárez, R. *et al.* A pan-mammalian map of interhemispheric brain connections predates the evolution of the corpus callosum. *Proc Natl Acad Sci U S A* **115**, 9622-9627 (2018).
3. García-Moreno, F. & Molnár, Z. Subset of early radial glial progenitors that contribute to the development of callosal neurons is absent from avian brain. *Proc Natl Acad Sci U S A* **112**, E5058-E5067 (2015).
4. Ako, R. *et al.* Simultaneous visualization of multiple neuronal properties with single-cell resolution in the living rodent brain. *Molecular and Cellular Neuroscience* **48**, 246-257 (2011).
